# Damage mapping techniques and the light they have shed on canonical and atypical UV photoproducts

**DOI:** 10.3389/fgene.2022.1102593

**Published:** 2023-01-10

**Authors:** Kaitlynne A. Bohm, John J. Wyrick

**Affiliations:** School of Molecular Biosciences, Washington State University, Pullman, WA, United States

**Keywords:** CPD, 6-4 photoproduct, transcription factor, nucleosome, thymine-adenine photoproduct, hotspot, mutation, melanoma

## Abstract

Ultraviolet (UV) light is a pervasive threat to the DNA of terrestrial organisms. UV light induces helix-distorting DNA lesions, primarily cyclobutane pyrimidine dimers (CPDs) that form between neighboring pyrimidine bases. Unrepaired CPD lesions cause cytosine-to-thymine (C>T) substitutions in dipyrimidine sequences, which is the predominant mutation class in skin cancer genomes. However, many driver mutations in melanoma (*e.g.,* in the *BRAF* and *NRAS* oncogenes) do not fit this UV mutation signature. Recent studies have brought to light the intriguing hypothesis that these driver mutations may be induced by infrequent or atypical UV photoproducts, including pyrimidine 6-4 pyrimidone photoproducts (6-4PP) and thymine-adenine (TA) photoproducts. Here, we review innovative methods for mapping both canonical and atypical UV-induced photoproducts across the genome.

## 1 Introduction

Ultraviolet (UV) radiation from the Sun is a pervasive threat to the genomic integrity of terrestrial organisms, including humans. UV-induced DNA damage may lead to mutations that give rise to both melanoma and non-melanoma skin cancers, the former of which accounts for a majority of skin cancer-related deaths (Saginala et al., 2021). Recent advances in sequencing technologies have afforded us the opportunity to take a closer look at the genome-wide distribution of UV-induced lesions responsible for skin carcinogenesis. UV-induced CPD lesions lead to C>T mutations in lesion-forming dipyrimidine sequences, which comprise the majority of somatic mutations in melanoma and other skin cancers (Hodis et al., 2012; Brash 2015). Intriguingly, these UV-induced C>T mutations are not responsible for many of the key driver mutations that cause melanomagenesis, including mutations in the *BRAF* (*i.e.,* V600E and V600K) and *NRAS* (*i.e.,* Q61R, Q61K, and Q61L) oncogenes (Hodis et al., 2012). Instead, mutation classes with little to no prior link to UV-induced DNA damage, including T>C, T>A, and AC>TT, are the culprits (Hayward et al., 2017; Laughery et al., 2020). This highlights the need to further investigate the intricate relationship between UV exposure, DNA damage, and mutagenesis, with a particular emphasis on rare UV photoproducts and oxidative damage. Recently developed methods have mapped different classes of UV photoproducts at (or near) single nucleotide resolution. Here we discuss key advances made in the field of UV-induced damage mapping, and how knowledge of this UV DNA damage spectrum can advance our understanding of UV mutagenesis in skin cancers.

### 1.1 Foundational studies

#### 1.1.1 CPD mapping at the nucleosome and promoter-level

Current high-resolution damage mapping technologies would not be where they are today if not for the foundational work that established our basic understanding of UV-induced DNA damage within chromatin. Gale et al. (1987) published the first UV damage map of CPDs within nucleosomes, the primary building block of chromatin. The authors UV-irradiated chromatin fibers, mononucleosomes, and intact mammalian cells and used the proofreading exonuclease activity of T4 DNA polymerase to map UV photoproducts, which are exonuclease-resistant (Doetsch et al., 1985), in nucleosomal DNA. Analysis of UV photoproducts in micrococcal nuclease (MNase) digested fragments revealed a striking 10.3 base pair (bp) periodicity within nucleosomes, in which CPDs preferentially formed at positions where the minor groove of DNA faced ‘out’ from histone octamers. This was one of the first indications that the packaging of DNA into chromatin significantly affects the UV damage landscape (Gale et al., 1987).

Becker and Wang (1984) and Pfeifer et al. (1992) were among the first to examine how transcription factor (TF) binding affects UV photoproduct formation. (Pfeifer et al., 1992) exploited the propensity of T4 endonuclease V (T4 endoV) to cleave DNA at CPDs, followed by ligation-mediated PCR (LM-PCR) to map cleavage events in the promoter region of the *PGK1* gene in UV-irradiated cells. A similar method used piperidine to map 6-4PPs. Their data indicated that CPDs and 6-4PPs were significantly modulated at a CCAAT sequence in the *PGK1* promoter, which serves as a consensus binding sequence for the NFY family of TFs. 6-4PPs were enriched at the ‘CC’ on the motif strand, while a CPD hotspot was identified at the ‘TT’ (opposite ‘AA’) on the non-motif strand. Conversely, certain positions within this promoter region exhibited reduced or ‘negative’ photofootprints. This, along with other studies (Becker and Wang 1984; Selleck and Majors, 1987), were foundational in introducing the idea that DNA binding by TFs could modulate UV damage formation Pfeifer et al. (1992).

#### 1.1.2 Microarrays expand the scope of CPD mapping

The first genome-wide map of UV damage was generated by Simon Reed and colleagues (Teng et al., 2011). Their innovative approach used the chromatin immunoprecipitation-microarray method (ChIP-chip), which had previously been used to map histone modifications and TF binding sites (Ren, Robert, Wyrick et al., 2000; Kurdistani et al., 2004), to instead map UV-induced CPD lesions. This was accomplished by employing an anti-CPD antibody in the chromatin immunoprecipitation step. The authors saw a strong correlation between measured and expected CPDs (based on DNA sequence context). This study was able to elucidate large-scale damage and repair patterns in genic versus intergenic regions, but its relatively low resolution (Table 1) rendered it unable to elucidate damage patterns within and around other protein-bound DNA sites.

**TABLE 1:** Methods described in this mini review, including type of UV damage mapped, method of damage detection, and sensitivity of each assay.

Method	Damage mapped	Method of detection	Mapping resolution
ChIP-chip Teng et al. (2011)	CPDs	Anti-CPD IP + microarray hybridization	∼400 base pairs (sonicated DNA fragment size)
ChIP-chip Zavala et al. (2014)	CPDs	Anti-CPD IP + microarray hybridization	∼300 base pairs (sonicated DNA fragment size)
ChIP-seq	CPDs and 6-4PPs	Anti-CPD or anti-6-4PP IP + + NGS	>100 by (sonicated DNA fragment size)
HS-Damage-seq	CPDs and 6-4PPs	Anti-CPD or anti-6-4PP IP + DNA polymerase stalling + NGS	Single nucleotide
CPD-seq	CPDs	T4 endoV cleavage + NGS	Single nucleotide
Adduct-seq	CPDs	T4 endoV cleavage + NGS	Single nucleotide
Circle-seq	CPDs and deaminated CPDs	T4 endoV or UDG cleavage + NGS	Single base pair (does not specify damaged strand)
UVDE-seq	6-4PPs and TA-PPs	UVDE cleavage + NGS	Single nucleotide
Excision-seq	CPDs and 6-4PPs	UVDE cleavage + photolyase repair + NGS	Single nucleotide

IP, immunoprecipitation and NGS, Next Generation Sequencing.

Utilizing similar ChIP-chip technologies, Zavala et al. (2014) was the first study to examine CPD formation at a chromosome-scale within the human genome. CPD lesions were mapped in UV-irradiated human skin fibroblasts *via* DNA fragment pull down by an anti-CPD antibody and hybridization to microarrays tiling human chromosomes 1–6. Their results revealed CPD hotspots were frequently associated with repetitive SINE elements. Many of these CPD hotspots were associated with polydA tracts (polydT on the opposite strand), suggesting poly-pyrimidine tracts are particularly prone to CPD formation. This method was subsequently improved upon by using ChIP-sequencing (ChIP-seq), which has been used by the Morrison lab to map CPDs and 6-4PPs across the human genome (García-Nieto et al., 2017; Perez et al., 2021). The Morrison group reported that UV damage is elevated in nuclear lamin-associated heterochromatin regions, potentially due to their more UV-exposed localization in the nuclear periphery.

### 2.1 Necessary tools for the job

The resolution of DNA damage mapping methods was significantly improved by switching from microarray hybridization to high-throughput DNA sequencing methods. Some damage mapping techniques still utilize immunoprecipitation of damaged DNA, while others utilize substrate specificity of certain enzymes to precisely map UV lesions. Below we discuss different methods for high-resolution (*i.e.,* single nucleotide resolution) mapping of CPDs, 6-4PPs, and atypical lesions.

### 2.2 High-resolution mapping of CPDs

#### 2.2.1 Targeted enzyme cleavage of CPDs

Initial methods for high resolution UV damage mapping capitalized on the enzymatic specificity of T4 endoV to map CPDs. Mao and colleagues developed the CPD-seq method, which utilizes T4 endoV to map CPD formation and repair in *S. cerevisiae* and, in a follow-up study, human fibroblasts (Mao et al., 2016; Mao et al., 2018). CPD-seq, adapted from the emRiboSeq protocol (Ding et al., 2015), involves UV irradiating cells, isolating and sonicating genomic DNA (gDNA), and ligating a first adapter to the DNA fragment ends (Table 1). Following ligation, any remaining free 3’ hydroxyls (3’OH) are blocked by the addition of dideoxy nucleotides by terminal transferase. Ligated fragments are treated with T4 endoV and APE1 to cleave upstream of CPDs and create a free 3’OH group to which the second biotinylated adapter is ligated. Ligated DNA fragments are purified using streptavidin beads and PCR amplified. Alignment of the resulting CPD-seq reads to the reference genome provides a single nucleotide resolution map of CPD formation and repair.

CPD-seq has revealed that in nucleosomes, CPDs form more readily at positions in which the minor groove of DNA faces ‘out’ from histone proteins, resulting in periodic formation of CPDs in nucleosome DNA that is mirrored by somatic mutations in skin cancers (Mao et al., 2016; Brown et al., 2018; Pich et al., 2018). CPD-seq further reported that DNA-binding by certain TFs promotes the formation of CPD lesions at specific locations in their binding motifs. Two independent studies utilized the CPD-seq method to report that binding by members of the E26 transformation-specific (ETS) family induce CPD hotspots within their TTCCG-containing binding motif in human fibroblasts and melanoma cells (Elliott et al., 2018; Mao et al., 2018). A later study reported CPD hotspot formation within sites targeted by the CCCTC-binding factor (CTCF) (Sivapragasam et al., 2021). Notably, locations of CPD hotspots in ETS and CTCF binding sites coincide with (and potentially can explain) mutation hotspots seen in sequenced melanomas and other skin cancers (Elliott et al., 2018; Mao et al., 2018; Sivapragasam et al., 2021).

AdductSeq (Premi et al., 2019), like CPD-seq, relies on enzymatic cleavage (Table 1). Isolated gDNA is treated with USER enzyme to nick at abasic sites and uracils not within CPDs. Nicks are then 5’ dephosphorylated to avoid incorporation in subsequent library steps, offering a unique method of lowering background reads. DNA is treated with T4 endoV to cleave CPDs and then CPD photolyase is used to monomerize (i.e., remove the CPD lesion) the base on the 5’ end of the fragment so this 5’ end can be ligated to an adapter. Random priming DNA synthesis then generates dsDNA, which aids in ligation of the biotinylated adapter to the 5’ end of the fragment. Library preparation is completed with shearing by sonication and biotin purification, and sequenced. This study reported CPD hyperhotspots at ETS family binding sites, consistent with other studies (Elliott et al., 2018; Mao et al., 2018). A follow up study revealed that CPD hyperhotspots at ETS binding sites reproducibly occur in other skin cell types (i.e., keratinocytes), particularly at ETS binding sites in CpG islands associated with genes involved in RNA processing and translation (Garcia-Ruiz et al., 2022).

While cytosine bases in DNA spontaneously deaminate to uracil very infrequently, rates of cytosine deamination are orders-of-magnitude faster in CPD lesions (Cannistraro and Taylor. 2009; Cannistraro et al., 2015). Circle-damage-seq (Jin et al., 2021) was developed to map cytosine deamination events associated with CPD lesions in UV-irradiated human cells. This method circularizes DNA following sonication and end repair, then cleaves deaminated CPDs after photolyase treatment with uracil DNA glycosylase (UDG). DNA is nicked opposite the cleaved uracil using a ssDNA-specific S1 nuclease, resulting in DNA double strand break. Adapters are ligated to each DNA end, and divergent paired-end sequencing produces reads on either side of the damage site. The authors reported a strong match in the distribution of deaminated CPDs throughout the human genome to mutational signatures observed in melanoma genomes. However, because this method generates and sequences double strand breaks, it does not directly indicate the lesion-containing DNA strand that was cleaved (Table 1).

#### 2.2.2 Antibody-based mapping of CPDs

An alternative method of detecting UV-induced CPDs involves immunoprecipitating DNA fragments that contain CPD lesions. HS-Damage-seq developed by the Sancar group uses CPD immunoprecipitation to enrich for CPD-containing DNA fragments, followed by primer extension by DNA polymerase to map lesions at high resolution (Hu et al., 2017). Briefly, DNA from UV-irradiated cells is isolated, sonicated, and undergoes double-stranded adapter ligation. This is followed by immunoprecipitation of the damaged strand by anti-CPD antibodies (or anti-6-4PP antibodies, discussed below). Primer extension is performed using biotin-containing primers, which is blocked by UV photoproducts. Unbiotinylated samples are removed, subtractive hybridization eliminates fragments without damage, and a final adapter is ligated to the end of the primer extension product to enable sample amplification and sequencing. The CPD lesion site is located just beyond the primer extension product, where the second adapter was ligated. Utilizing primer extension to map DNA lesions allows for single-nucleotide resolution mapping of a variety of different lesion classes. While the lesion-specific immunoprecipitation step is critical for the success of this method, it can potentially introduce biases, as anti-CPD antibodies can have sequence preferences (*e.g.,* higher affinity for TT CPDs, etc.) (Mori et al., 1991). However, a recent study indicated that the primary bias introduced by anti-CPD and 6-4PP antibodies is a potential under-representation of lesions at CC CPDs or 6-4PPs (Wu et al., 2022).

HS-damage-seq has demonstrated that numerous transcription factors modulate CPD and 6-4PP formation in human cells. The authors found that CPDs are enriched at NFYB and POU2F2 binding sites in UV-irradiated cellular samples compared to naked DNA, the former consistent with previous findings (Pfeifer et al., 1992). This same group developed a similar assay in which NER repair fragments can be sequenced (XR-seq; Hu et al., 2015; Adar et al., 2016) using a similar antibody pulldown step and ligation-mediated DNA sequencing. This method has since been refined (ALT-XR-seq; Wu et al., 2022) to replace lesion immunoprecipitation with photolyase repair and PCR, and reports similar damage findings to XR-seq, with the possible exception of lesions occurring at CC dinucleotides (see above). The data accumulated from both HS-Damage-seq and XR-seq provide powerful tools utilized by many groups to study damage and repair in a variety of genomic contexts (Perera et al., 2016; Sabarinathan et al., 2016; Mao et al., 2018; Pich et al., 2018; Perez et al., 2021).

### 3.1 Genome-wide mapping the entire spectrum of UV photoproducts

While most studies have focused on mapping CPDs, previous studies have suggested that 6-4PPs and atypical UV photoproducts may have important roles in UV mutagenesis (Bresson and Fuchs 2002; Laughery et al., 2020). Here, we discuss genome-wide methods used to map these rare UV photoproducts.

#### 3.1.1 Mapping 6-4 photoproducts

The first method to map 6-4PPs across the genome was Excision-seq developed by Hessebreth and colleagues (Bryan et al., 2014). They utilized the broad substrate specificity of the *S. pombe* Uve1 (also known as UVDE; ultraviolet damage endonuclease) enzyme, which can cleave at both CPDs and 6-4PPs, to map UV photoproducts across the yeast genome following irradiation with 10,000 J/m^2^ UVC. This high dose was imperative to the experimental design, as the assay relied on multiple UV photoproducts forming in close proximity so cleavage would release a small dsDNA fragment for subsequent adapter ligation and sequencing. Following cleavage by Uve1, samples were treated with either CPD or 6-4 photolyases to map CPDs or 6-4PPs, respectively. Photolyase treatment removes the photoproduct associated with the 5’end of the Uve1 cleavage site to allow for end polishing and adapter ligation. The authors reported a roughly uniform distribution of CPDs and 6-4PPs; however, the effects of nucleosomes or TFs on photoproduct formation was not analyzed.

Our group recently adapted the CPD-seq protocol to accurately map the location of 6-4PPs throughout the yeast genome (Laughery et al., 2020; Sivapragasam et al., 2021; Bohm et al., 2022) in a method we termed UVDE-seq. This method utilizes the repair specificity of *E. coli* CPD photolyase and the broad cleavage abilities of *T. thermophilus* UVDE to map 6-4PPs and atypical TA-PPs. Although further studies are needed to examine non-CPD lesion formation within chromatin, this method offers an exciting new avenue in which to study the formation of less frequently formed photoproducts.

The HS-Damage-seq assay offered the first look at the formation and repair of 6-4PPs across the human genome and over an extended repair time course (Hu et al., 2017). Coupled with XR-seq repair data, the authors suggested UV photoproduct formation is mostly influenced by DNA sequence context, while repair is modulated by chromatin. However, they did find that DNA binding by certain TFs (*e.g.,* NFYB, etc.) modulated UV photoproduct formation. Subsequent bioinformatics analysis of HS-Damage-seq data by the Lopez-Bigas group revealed that 6–4PPs are elevated at minor-out positions within nucleosomal DNA (Pich et al., 2018).

ChIP-seq using anti-6-4PP antibody has also been used to map 6-4PP formation across the human genome (Perez et al., 2021). The authors found that large-scale patterns of 6-4PP susceptibility closely mirrored that of CPDs, showing enrichment within heterochromatin and transcriptionally repressed regions. They also reported several oncogenes and tumor suppressors with higher mutagenic potential (defined as susceptibility to damage/repair ratio), including *NRAS*.

#### 3.1.2 Discovery and genome-wide maps of thymine-adenine photoproducts

The existence of a UV-induced thymine-adenine photoproduct (TA-PP) was discovered nearly 40 years ago (Bose et al., 1983). Following its discovery, studies performed in *E. coli* determined these lesions to be highly mutable, frequently resulting in A>T mutations (Zhao and Taylor 1996; Otoshi et al., 2001). TA-PPs are suggested to occur ∼10–100-fold less frequently than TT dimers (Otoshi et al., 2001; Davies et al., 2007), potentially complicating our ability to map these lesions within whole genomes. Excision-seq offered the first high-resolution, genome-wide look at UV damage associated with TA dinucleotides. However, it was not clear from this study whether lesions associated with TA sequences were *bona fide* UV photoproducts or experimental artifacts produced by photolyase bias (Bryan et al., 2014).

A recent study from our group added validity to these TA reads, as *in vitro* analysis demonstrated induction of TA-PPs following UVC exposure, and the newly developed UVDE-seq assay mapped TA-PPs across the entire yeast genome, at a frequency higher than canonical 6-4PPs at some sequence contexts. UVDE has a broad substrate specificity and recognizes both helix-distorting CPDs and 6-4PPs, which may explain why UVDE is able to recognize a similarly helix-distorting TA-PP. This same study suggested up to 12% of UV-induced mutations may arise from mutagenic bypass of TA-PPs, emphasizing the need to further investigate these lesions and their potential role in mutagenesis of the eukaryotic genome (Laughery et al., 2020).

## 4.1 Discussion

**FIGURE 1:**
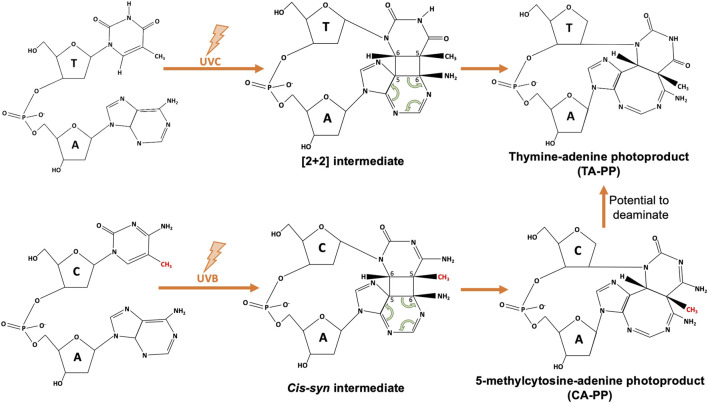
Schematic of atypical thymine-adenine (TA-PP) and cytosine-adenine photoproduct (CA-PP) formation. Upon UVC exposure, neighboring nitrogenous bases of a 5’ thymine and 3’ adenine will form covalent bonds at C5 and C6 to form an unstable [2 + 2] intermediate structure, that finalizes as the TA-PP (structure adapted from Wang et al., 2001). Similarly, as discussed in Su et al. (2010), methylated cytosines (methyl group shown in red) within a 5’-^m^CA-3’ context have the ability to form a CA-PP following UVB exposure, that then may deaminate to form a TA-PP.

While we know the main players involved in UV damage formation, the full spectrum of UV-induced DNA lesions is still being uncovered. Non-canonical mutations seen in skin cancers without a clear causative lesion include AC > NN and CA > NN tandem mutations (Laughery et al., 2020). While it is possible that non-canonical mutations such as these could arise from mutagenic bypass of a nearby lesion, AC > NN mutations do not follow a specific consensus flanking sequence, suggesting the AC could be a photoproduct itself (Laughery et al., 2020). AC>TT, AC>CT, CA>TT, and CA>AT mutations can activate the *BRAF* oncogene in melanomas; however, data showing the induction of AC or CA photoproducts (Figure 1) is limited (Su et al., 2010), and even less is known about the frequency or context of such lesions. Additionally, the presence of a TG lesion has been suggested, although its source and molecular identity is unclear. AdductSeq data suggests TG reads may be associated with oxidation events resulting from melanin production (Premi et al., 2019), while work by the Nikolaev group suggests a UV-induced TG lesion may contribute to G>T mutations in XP-variant human skin cancers (Yurchenko et al., 2022). Optimization of the damage mapping techniques discussed above may determine if this is in fact a UV-induced photoproduct or a result of UV-induced base oxidation.

Continued advancement of these damage-mapping technologies should improve our understanding of damage formation patterns throughout the human genome, and provide new insights into the mutation spectra seen in skin cancers.
